# Does Right‐Hand Motor Impairment Affect Performance on Cognitive Testing in Parkinson's Disease?

**DOI:** 10.1002/mdc3.70178

**Published:** 2025-06-09

**Authors:** Priyanka Rao, Christine A. Cooper, Travis H. Turner

**Affiliations:** ^1^ College of Medicine Medical University of South Carolina Charleston South Carolina USA; ^2^ Department of Neurology Medical University of South Carolina Charleston South Carolina USA

**Keywords:** Parkinson's disease, laterality, motor impairment, cognitive testing, graphomotor

## Abstract

**Background:**

For People with Parkinson's (PwP), motor disturbances affecting the dominant upper extremity often impacts ability to perform tasks such as writing or typing and may confound cognitive test performances with graphomotor responding.

**Objectives:**

We explored association between upper extremity impairment and graphomotor cognitive test performances in a clinical sample of moderately advanced PwP pursuing Deep Brain Stimulation (DBS).

**Methods:**

A retrospective chart review was conducted of 80 consecutive right hand dominant PwP who completed pre‐DBS neuropsychological evaluations. Right upper extremity (RUE) impairment was measured using MDS‐UPDRS Part 3. Non‐parametric correlations examined associations between RUE impairment and tests involving graphomotor (trail making, letter cancellation, visual memory) versus oral responding (fluency, digit span, verbal memory).

**Results:**

No statistically significant associations were observed between RUE motor impairment and graphomotor test performances.

**Conclusions:**

Minimal impact of dominant RUE impairment on graphomotor tasks in moderately advanced PwP supports application in clinical and research settings.

Beyond the hallmark movement disturbances, Parkinson's disease (PD) can manifest psychiatric, cognitive, sensoriperceptual, and autonomic symptoms.^1^ The underlying basis for a functional problem that a Person with Parkinson's (PwP) experiences in daily life may reflect interactions amongst motor and non‐motor symptoms. For instance, struggles using a smartphone may reflect a combination of tremor, dexterity, and cognition, all of which are compounded by anxiety. Problems with driving might reflect cognition, visual perception, and bradykinesia.

Neuropsychological evaluations can be useful in assessing presence and severity of cognitive deficits and psychiatric symptoms and disentangling their contributions to functional limitations. However, many standardized cognitive tests require graphomotor responding, which may be confounded in PwP by motor symptoms such as bradykinesia, dyspraxia, and tremor. Published literature has advised caution when administering tests requiring graphomotor responses with people with PD due to potential impact of motor symptoms on test performance.^2^, ^3^, ^4^ In a study involving newly diagnosed, untreated PwP from the Parkinson's Progression Markers Initiative (PPMI) cohort,^5^ the only neuropsychological test where cognitively normal PwP performed worse than healthy controls was the Symbol‐Digit Modalities Test (SDMT),^6^ a timed measure involving writing numbers to match symbols; however, this difference was no longer significant after controlling for motor impairment in the dominant right upper extremity (RUE).^7^

Understanding impact of motor impairments on cognitive test performance is essential for accurate clinical evaluations and treatment planning. This is particularly relevant for patients undergoing advanced therapies such as deep brain stimulation (DBS), where candidacy is evaluated using neuropsychological tests, and motoric confounds affecting test performances could impact treatment planning and symptom monitoring. Accordingly, we use only three tests in our clinical battery that require graphomotor responding: the Trail Making Test (TMT)^8^ for executive functioning, the Neuropsychological Assessment Battery (NAB)^9^ Numbers & Letters, Condition A, for sustained attention and vigilance, and the Brief Visuospatial Memory Test (BVMT‐R)^10^ for visual memory. These tests were selected as they provide important information regarding aspects of cognitive functioning that cannot be easily replaced with non‐motor equivalents and have less fine manual dexterity demands than the SDMT. The oral version of the SDMT was not included as it has not been validated in PD. In this study we examined whether severity of motor impairment to the dominant RUE was associated with worse performance on these standardized graphomotor cognitive tests administered during pre‐DBS neuropsychological evaluations.

## Methods

A retrospective chart review was performed with approval from our human subjects protection program (PRO: 00062817) on a cohort of consecutive PwP who completed comprehensive neuropsychological evaluations of initial candidacy for Deep Brain Stimulation (DBS) and had complete Movement Disorder Society‐Unified Parkinson's Disease Rating Scale (MDS‐UPDRS)^11^ Part 3 (motor examination) ratings from a fellowship‐trained movement disorder neurologist. Hand dominance was self‐reported and based on writing; left‐hand dominant and ambidextrous patients were excluded to reduce potential impact of atypical lateralization of cerebral function. To best align motor functioning with cognitive test performance, only patients with complete MDS‐UPDRS Part 3 examinations performed during the subjective medication “on” state prior to surgery and within 3 months of neuropsychological evaluation were included. When available, contemporaneous MDS‐UPDRS Part 3 ratings during the “off” condition were also extracted. Neuropsychological evaluations were completed or supervised by the senior author during the subjective “on” state. Levodopa equivalent daily dose (LEDD) was documented at time of neuropsychological evaluation.^12^

Motor impairment for RUE and LUE were estimated by the sum of MDS‐UPDRS Part 3 ratings from rigidity (3.3), finger tapping (3.4), hand movements (3.5), pronation and supination (3.6), postural tremor (3.15), kinetic tremor (3.16), and rest tremor (3.17). These scores were summed allowing for a total score ranging between 0 and 28. All graphomotor measures from the neuropsychological battery were examined, including the TMT (Trails A Time and Trials B Time),^8^ NAB Numbers & Letters condition A (Time and Errors),^9^ and the BVMT‐R (form 1, Total Learning and Delay Recall).^10^ Oral response measures included the Controlled Oral Word Association Test (Phonemic Fluency and Semantic Fluency),^8^ NAB Digit Span (Forward and Backward),^9^ and the Hopkins Verbal Learning Test, revised (HVLT‐R, form 1, Total Learning and Delay Recall).^13^ Raw scores for neuropsychological measures were transformed to T‐scores (mean = 50, SD = 10) adjusting for demographics according to test manuals.

Associations between RUE and LUE motor impairment and cognitive test performances were examined using Spearman‐Brown rank‐order correlations (rs). The Bonferroni approach was used to control for false discovery rate (six measures for each response modality, *p* < 0.0083). All statistical analyses were performed using SPSS® Version 26.

## Results

Complete MDS‐UPDRS Part 3 “on” motor examinations were obtained from 80 right‐hand dominant PwP seeking DBS. Clinical and demographic are provided in Table 1. The majority were male (74%) ranging in age from 42 to 81 years with a mean (SD) duration of illness of 9.60 (4.42) years. One patient was taking 400 mg LEDD medication at time of MDS‐UPDRS motor examination but had discontinued all medication prior to neuropsychological evaluation. Another patient was only taking primidone for refractory tremor and no dopaminergic medications during both motor and neuropsychological examinations. About one‐third of the sample had initial symptom onset on the right side (*n* = 30, 38%), about half had symptom onset on the left (*n* = 37, 46%), with the remainder (*n* = 13, 16%) not having clearly lateralized symptom onset. Mean (SD) severity of motor impairment in the sample was similar for RUE, 6.25 (4.13) and LUE 6.26 (3.71). About three‐quarters of the sample (*n* = 58) had MDS‐UPDRS Part 3 examinations performed during the “off” state, prior to “on” examination, with a typical reduction in motor impairment of about 33% following levodopa treatment. Mean (SD) performance on a global cognitive screen (Mattis Dementia Rating Scale)^14^ was within the normal range, 139.74 (4.27). Applying Movement Disorders Society Task Force Level II diagnostic criteria for Mild Cognitive Impairment in Parkinson's disease^15^ and Parkinson's disease dementia^16^ two PwP were diagnosed with dementia (2%) and 43 (54%) were diagnosed with mild cognitive impairment.

**TABLE 1:** Clinical and demographic characteristics

Measure	Mean (SD)	Min	Median	Max
Age	64.78 (8.46)	42	65	81
Gender (% men)	59 (74%)			
Education (years)	14.99 (2.94)	8	16	20
Duration of illness (years)	9.60 (4.42)	2	9	21
LEDD (mg)	1137 (567)	0	1122	3070
DRS‐2 total score (raw)	139.74 (4.27)	123	141	144
MDS‐UPDRS Part 3 total (On)	28.24 (11.87)	7	26	62
MDS‐UPDRS Part 3 RUE	6.25 (4.13)	0	5	20
MDS‐UPDRS Part 3 LUE	6.26 (3.71)	0	6	17
MDS‐UPDRS Part 3 total (Off)^a^	43.29 (12.89)	18	42	78
MDS‐UPDRS Part 3 On vs. Off^a^	16.29 (9.58)	0	14.5	51

^a^ MDS‐UPDRS Part 3 scores were available for a subset of *n* = 58 PwP (73% of full sample).

As shown in Table 2, RUE impairment was not associated with any of the motor measures included in the test battery. Inclusion of LEDD as a covariate did not change these results. However, a statistically significant correlation was found between RUE impairment and one of the oral response measures, phonemic fluency (rs = −0.299, *p* = 0.008). Weak correlations between RUE impairment and the oral response measure of digits backward, rs = −0.276, *p* = 0.013, and LUE impairment with motor response measures of Numbers & Letters A Time, rs = −0.282, *p* = 0.031, and BVMT Total Learning, rs = −0.248, *p* = 0.03, fell short of statistical significance when accounting for multiple comparisons. In exploratory follow‐up analyses, inclusion of LEDD and effect of levodopa (Off–On) did not reveal statistically significant correlations between RUE impairment and graphomotor test scores.

**TABLE 2:** Association between upper extremity motor impairment and neuropsychological test performances

	Measure	*n*	Mean (SD)	RUE	LUE
Motor response	Trails A	73	48.78 (11.60)	−0.138	−0.217
	Trails B	72	44.47 (12.57)	−0.041	−0.014
	Number & letters a time	59	43.92 (12.06)	−0.098	−0.282*
	Number & letters a errors	59	47.75 (11.31)	−0.137	−0.200
	BVMT total learning	72	41.18 (11.65)	−0.191	−0.248*
	BVMT delayed recall	72	44.01 (12.38)	−0.058	−0.231
Verbal response	Phonemic fluency (FAS)	78	47.78 (10.44)	−0.299**	−0.195
	Semantic fluency (animals)	78	49.79 (10.43)	−0.106	−0.151
	Digits span forward	80	51.51 (9.33)	−0.206	−0.047
	Digit span backward	80	48.16 (8.77)	−0.276*	−0.190
	HVLT total learning	77	40.90 (10.71)	−0.096	0.028
	HVLT delay recall	77	38.79 (11.51)	−0.118	−0.076

* Significant at *p* < 0.05 level.

** Significant at *p* < 0.01 level.

## Discussion

Results from this study found that motor impairment to the dominant RUE was not significantly associated with worse performance on several commonly used neuropsychological tests involving graphomotor output. This finding stands in contrast to generalized admonitions about using such measures in evaluations of PwP, and a previous observation by our group of an association between RUE motor impairment in newly diagnosed PwP.^4^ One reason for the discrepancy is that participants in the prior study were not symptomatically treated at time of evaluation, and levodopa therapy may ameliorate impact of motor deficits on testing. Additionally, the graphomotor demands between the tasks used in the current study, that is, connecting dots, striking through Xs, and untimed rendering of basic figures, requires less fine manual dexterity than the SDMT from the previous study, which requires quick writing of numbers. A weak correlation was found between phonemic verbal fluency and RUE impairment. While not the focus of this investigation, this finding is not surprising given left lateralization of language and previous studies in PD demonstrating similar associations.^17^, ^18^, ^19^

Restriction of neuropsychological batteries to tests without graphomotor demands limits the clinician's ability to assess domains of visuospatial memory, executive, and visuospatial function. For instance, a recent multicenter study of PwP undergoing DBS found that presurgical deficits in visual recall (BVMT) and set‐switching (TMT) uniquely predicted postsurgical multidomain cognitive decline.^20^ Another study found that visual recall was highly sensitive to unsafe driving in PwP.^21^ Rather than eliminate graphomotor measures altogether, we advocate for thoughtful consideration of the nature of motor impairment (eg, fine versus gross) and the clinical question. This may require addition of complementary oral or untimed graphomotor measures, and a nuanced interpretation of scores that considers impact of motor function on performance.

The null finding in our study may reflect limited statistical power. However, none of the expected associations even showed a trend toward statistical significance, suggesting negligible clinical relevance at the individual level, As discussed above, these findings do not extend to early untreated PD or advanced PD, or timed tasks requiring fine manual dexterity such as the SDMT.^3^ Dyskinesia, a common medication complication in PwP considering DBS, was also not examined as a potential confound. Additionally, as these tasks were done by patients on symptomatic therapy, findings cannot be extrapolated to testing while off medication. A multicenter study is currently underway that will examine impact of motor impairment to the dominant RUE on the SDMT in moderately advanced treated PD.

## Author Roles

(1) Research project: A. Conception, B. Organization, C. Execution; (2) Statistical Analysis: A. Design, B. Execution, C. Review and Critique; (3) Manuscript Preparation: A. Writing of the first draft, B. Review and Critique.

P.R.: 1A, 1C, 2B, 3A.

C.A.C.: 1A, 2C, 3B.

T.H.T.: 1A, 1B, 2A, 2B, 2C, 3B.

## Disclosures

**Ethical Compliance Statement:** This study was approved by the Institutional Review Board for Human Research at the Medical University of South Carolina, Pro00062817. This study was a retrospective chart review at an academic medical center; informed consent was not required. We confirm that we have read the Journal's position on issues involved in ethical publication and affirm that this work is consistent with those guidelines.

**Funding Sources and Conflict of Interest:** No specific funding exists for this work. The authors declare that there are no funding sources or conflicts of interest relevant to this work.

**Financial Disclosures for the Previous 12 Months (None Relevant to Current Work):** PR: Ms. Rao has no financial disclosures. CAC: Dr. Cooper serves on the Scientific Advisory Board for Abbvie. THT: Dr. Turner has received financial compensation for consultation to WCG Clinical Science and Scion Neurostim.

## Data Availability

The data that support the findings of this study are available on request from the corresponding author. The data are not publicly available due to privacy or ethical restrictions.
